# Innovative Approaches to Increase Access to Medicines in Developing Countries

**DOI:** 10.3389/fmed.2017.00218

**Published:** 2017-12-07

**Authors:** Hilde Stevens, Isabelle Huys

**Affiliations:** ^1^Institute for Interdisciplinary Innovation in healthcare (I3h), Unversité libre de Bruxelles (ULB), Brussels, Belgium; ^2^Clinical Pharmacology and Pharmacotherapy, Department of Pharmaceutical and Pharmacological Sciences, KU Leuven, Leuven, Belgium

**Keywords:** public–private partnership, product development partnership, intellectual property, pricing mechanism, access to healthcare

## Abstract

Access to essential medicines is problematic for one third of all persons worldwide. The price of many medicines (i.e., drugs, vaccines, and diagnostics) is unaffordable to the majority of the population in need, especially in least-developed countries, but also increasingly in middle-income countries. Several innovative approaches, based on partnerships, intellectual property, and pricing, are used to stimulate innovation, promote healthcare delivery, and reduce global health disparities. No single approach suffices, and therefore stakeholders need to further engage in partnerships promoting knowledge and technology transfer in assuring essential medicines to be manufactured, authorized, and distributed in low- and middle-income countries (LMICs) in an effort of making them available at affordable and acceptable conditions.

## Introduction

Today’s healthcare systems, both in developed and developing countries, face serious challenges. And time is pressing; in the years to come, we will face a dramatic shift in health problems resulting from epidemiological transition (1, 2). The poorest countries in developing regions carry the highest burden of disease: communicable diseases (CDs), non-communicable diseases (NCDs) (3), and the risk of new diseases related to changes in the social and physical environment, the socio-behavioral illness (4).

Access to essential medicines is problematic for one third of all persons worldwide (5). The price of many medicines (i.e., drugs, vaccines, and diagnostics) is unaffordable to the population in need, especially in the least-developed countries, but also increasingly in middle-income countries. The latter category comprises 105 countries, accounting for 70% of the world population, 75% of the poor, and a majority of the global disease burden (6, 7). Prices may decrease when multiple companies need to share market, in which context overcoming intellectual property (IP) obstacles is essential (8).

Stakeholders bundle forces in assuring essential medicines are manufactured, authorized, and distributed in low- and middle-income countries (LMICs) at affordable conditions. But challenges remain, i.e., guaranteeing high distribution coverage, ensuring affordability, and adoption of essential medicines, both at provider level and end-user level (9). Developing countries lack infrastructure needed to increase access to medicines. Most diagnostics are not designed for implementation in non-optimal laboratory conditions present in developing countries, with lack of air conditioning, stable electrical power, or refrigerators to store samples and chemicals (10, 11). Through microfluidic systems, high-tech technologies could find their way to the developing world laboratories. But the need for faster and more accurate diagnostics remains (10).

This article provides an overview of innovative approaches by stakeholders to address health challenges in developing countries, shedding light on business models for healthcare delivery. We look at the role of partnerships, IP, and specific pricing models for promoting innovation by providing incentives to invest in (collaborative) research and development (R&D), as well as to increase access to medicines.

## Method

A comprehensive review of literature was performed. Relevant articles were identified by searching databases, such as PubMed, Google Scholar, and documents like Official Journal of the European Union (EUR-Lex) up to March 2016, with an update in September 2017. Websites of relevant organizations, including public-private partnerships (PPPs) such as the innovative medicines initiative (IMI) and the medicines for malaria venture (MMV), pharmaceutical organizations’ websites, such as GlaxoSmithKline (GSK) and European Federation of Pharmaceutical Industries and Associations (EFPIA), and non-governmental organizations’ websites and private foundations’ websites, such as World Health Organization (WHO), World Intellectual Property Organization (WIPO), World Trade Organization (WTO), and Bill & Melinda Gates Foundation (BMGF), were explored.

## Partnerships as a Model to Facilitate Access to Medicines

Pharmaceutical companies no longer stick to traditional drug development models to tackle the enormous health challenges ahead of us. Being the key player in the drug development process, the pharmaceutical industry is partially responsible for finding solutions. During the last decades, the majority of the 20 largest research-based pharmaceutical companies have increased efforts to provide access to essential medicines in developing countries (12), e.g., by supporting or participating in product development partnerships (PDPs) (9).

Historically, PDPs directed toward neglected tropical diseases (NTDs) were the first collaborative efforts to tackle inequities in the health sector. In 1987, Merck & Co. donated ivermectin (Mectizan^®^) to treat onchocerciasis or river blindness, first distributed by the African Programme for Onchocerciasis Control (APOC), a partnership between the World Bank, the WHO, and non-governmental organizations (NGOs) in West-Africa, and expanded later to Africa and America (13, 14).

Product development partnerships, such as the MMV, have served as a source of inspiration for the pharmaceutical industry to apply the PPP model to disease areas other than NTDs, such as NCDs. The IMI, driven by EFPIA and supported by the European Commission (EC), has been a flagship early-phase research PPP (15). Initially, IMI focused on NCDs, but as the PPP matures, it aims at tackling NTDs (14). The European Lead Factory (ELF), for example, explicitly waives certain fees related to non-profit drug discovery programs for NTDs (16).

Public–private partnerships, and PDPs in particular, are vehicles suitable for delivering healthcare and strengthening healthcare systems. Such multi-stakeholder efforts are able to ensure product registration, increase local production and distribution capacity, and ensure governance for global health, e.g., adoption of new health technologies in national treatment policies in disease-endemic countries. In this way, PDPs such as the MMV, Drugs for Neglected Diseases Initiative (DNDi), and Institute for One World Health (OWH), advance public health (17, 18). PDPs strengthen research capacity in LMICs by building infrastructures at trial sites, providing equipment and setting up training in good clinical practice (GCP) and dedicated disease-specific research platforms in endemic countries. To achieve its objectives, PDPs partner with different stakeholders, such as high-income country pharmaceutical companies, local manufacturers, national disease-specific control programs and platforms, national governments and philanthropic organizations (9).

Public–private partnerships leverage knowledge and technology transfer of new medical technologies to both developed and developing countries. For example, the mobile health application (mHealth) Text4Baby, providing free health information to expectant mothers by means of text messages, is a PPP that, through a network of hundreds of partners, scales up its services (19). Partnerships could improve the scale of knowledge and technology transfer capacity of African Institutions that prove to be leaders in their area of focus (20). PPPs can improve both health products and services delivery by scaling their programs to a national level, involving health workers and communities (21). mHealth strategies are linked to improved data collection and reporting, planning and decision-support, training, and overall improved communication (22).

Moreover, knowledge and technology transfer should happen in both directions. Completely rethinking business models or applying reverse innovation requires adequate examination of models applied in developing countries in the context of developed countries. The technology applied in low-resource programs can stimulate innovation in developed countries, for example, by using text messages and interactive voice recognition systems instead of smartphone applications or by targeting the markets of the underserved such as elderly or immigrant communities (19). In this way, an innovation continuum can exist.

## IP as a Tool to Facilitate Access to Medicines

A robust IP framework is perceived by most right holders as essential to guarantee contribution to the state of the art while maintaining control over how creations, protected by IP rights (IPRs), are used. As such, IPRs have a facilitating role for improved healthcare in developing countries and should not be perceived as a hindrance. In pharma, patents are considered as the most important IP protection tools, providing the owner exclusive rights to prevent use of the patented product or process without the consent of the owner for 20 years in a particular territory. Data protection and market exclusivity rights are IPRs granted to the market authorization (MA) holder for a period of 8, 10 (or 11) years, respectively, after MA. Generic or biosimilar products are not allowed to enter the market as long as such IPRs are in force.

In many cases, at the time of product launch on the market, at least half of the patent term may have expired. Accordingly, industry claims to prolong the period of protection for their medical inventions (23). In theory, an increase of the period for patent and data or market exclusivity can increase profits, which may lead to innovation if appropriately re-invested in R&D. However, there is no any evidence that innovation thrives when extending exclusivity terms. In addition, generics are relied upon by most developing countries. Potential consequences of implementing prolonged exclusivity periods in developing countries could be enormous. Some propose that revenues from extended patent terms could be considered as a source of funding for drug donations to the least-developed countries (24).

One major issue is to guarantee that patent protection for pharmaceutical products creates incentives for R&D and does not hinder patient access in developing countries. The Agreement on Trade-Related Aspects of Intellectual Property Rights (TRIPS) of the WTO, implemented in 2005, created on the one hand incentives for R&D by introducing minimum standards for protection of IP. On the other hand, it introduces some flexibility such as compulsory licenses whereby access to IP protected technologies is granted *via* licenses imposed by governments, based on specific criteria, for instance, public health reasons. The members at the Doha Ministerial Conference in November 2001 agreed upon exemptions to patent protection in least-developed countries till 2016, with a potential extension to 2033 (25, 26).

With respect to compulsory licensing, some LMICs, not able to produce new drugs, could invoke such licensing and hence rely on capabilities of a developed country. Some state that compulsory licensing may cause consequences on other markets, as lower pricing of a compulsory licensed drug may trigger parallel export of the cheaper drug into more expensive markets.

The issuing of compulsory licenses gives a certain level of autonomy to Southern countries, but implies a certain legal, administrative, and reputational cost. In addition, some firms believe that compulsory licensing diminishes their incentives for innovation (11). Nonetheless, the overall impact of compulsory licensing seems beneficial (27). The TRIPS Agreement created an environment legitimizing innovators and generic companies, stimulating cross-border alliances, increasing numbers of R&D alliances, patent filings, and R&D investment. The main issue remains the impact of TRIPS on drug pricing and on biopharmaceutical companies’ willingness to invest in health problems at the local level (28).

Measures included in trade and investment agreements, e.g., the much debated Transatlantic Trade and Investment Partnership (TTIP) or the Trans-Pacific Partnership (TPP) agreements, impose far-reaching IP standards favoring the monopoly of large drug producers: extending patent terms, lowering patentability criteria, data/market exclusivity preventing generic and biosimilar drugs to enter the market, data protection obligations for biologics enabling high prices of e.g., cancer biologic drugs to remain longer on the market and routes to block generic drugs from entering the country. These provisions, called the TRIPS-Plus provisions, can, when translated effectively into domestic law, disproportionately affect developing countries by leading to high prices for essential medicines. To this end, policy makers have a crucial role in negotiation of policies and regulations (29, 30).

Other IP mechanisms relevant in the access debate are patent pools. The medicines patent pool (MPP) aims to enable affordable production of HIV drugs still under patent protection by obtaining voluntary licenses from patent holders and making these licenses available to generic companies in LMICs. Royalties will be paid to patent holders, and licenses to generic companies will be offered only in LMICs (8).

Another initiative in this line is an intellectual property, technology and know-how (IPTK) bank, a single platform licensed as a package with associated training modules. The IPTK bank could offer assistance in navigating vaccine registration with national regulatory authorities. The licensing approach covers patented technology to be disseminated to multiple developing-countries vaccine makers and royalties paid to the patent holder. IPTK banks would require an initial period of funding until provision of affordable vaccines would render them to be self-sustainable (8). Currently, the main source of external funding for vaccines in low-income countries is the global alliance for vaccines and immunizations (GAVI) (11). The subsidies provided by GAVI to finance new vaccines in a response to the 2012 World Health Assembly Global Vaccine Action Plan are intended to be limited to a 5-year period, with the expectation that, over that time, prices will fall, allowing donors and national governments to continue vaccine financing. Unfortunately, to date, this expectation has not been realized. There remains a need to establish mechanisms ensuring sustainable vaccine pricing once the initial period of support has ended (8).

The WHO report “Research and Development to Meet Health Needs in Developing Countries” considered several policies/models for access to medicines. The first model is a global framework on R&D, supporting priority medical R&D aimed at addressing neglected diseases. This model is not meant to replace the current IPR system, but is an additional instrument to meet the R&D needs of developing countries (31). The second model is a proposal that deals with open approaches to R&D, such as open innovation, open source, open access publishing, precompetitive R&D platforms, and equitable licensing (32).

In both proposed models, there is a need for flexible application of IPRs to reduce IP hurdles for innovation, which may reduce duplication in research and contribute to capacity-building, knowledge, and technology transfer (32). The WHO also covers the ethical dimension and the myriad of economic, commercial, technological, and regulatory factors related to providing healthcare, particularly to the poor (33).

**Figure 1:**
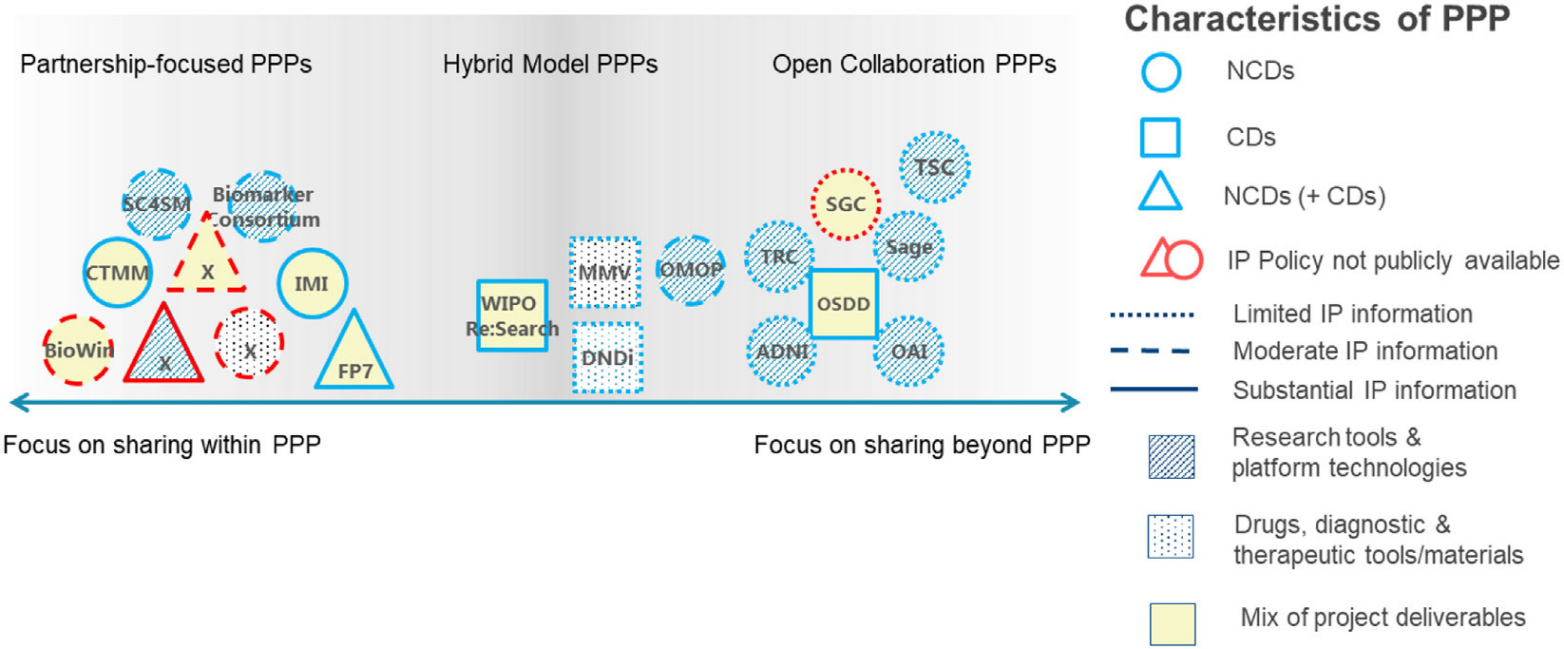
Link between intellectual property (IP) frameworks as defined in the IP policies of the public–private partnership (PPPs) analyzed, the information provided in the IP policies, project focus, and project deliverables. PPPs are categorized by research focus [non-communicable diseases (NCDs, circles), communicable diseases (CDs, squares), or a mix (triangles)]; availability of IP information [unavailable (gray outlines) and limited, partial, or substantial availability (black outlines)]; and deliverables [research tools and platform technologies (striped shading), drugs, diagnostic, and therapeutic tools or materials (dotted shading) or a mix (no shading)]. ADNI, Alzheimer’s Disease Neuroimaging Initiative; BioWin, Biotechnologies Wallonie Innovation; CTMM, Center for Translational Molecular Medicine; DNDi, Drugs for Neglected Diseases Initiative; FP7, European Framework Programmes; IMI, innovative medicines initiative; MMV, medicines for malaria venture; ND, not disclosed by PPP request; OAI, Osteoarthritis Initiative; OMOP, Observational Medical Outcomes Partnership; OSDD, Open Source Drug Discovery; SC4SM, Stem Cells for Safer Medicines; TSC, the SNP Consortium; TRC, the RNAi Consortium; SGC, Structural Genomics Consortium. [Figure adapted from Stevens et al. (34) with permission from the authors.]

Several types of IP strategies can be adopted in these two models, depending on the type of partnership. In partnerships focused on CDs, a partnership-focused strategy is adopted whereby IP is preserved for project partners. In partnerships focused on developing diagnostic devices or on NCDs, often an open collaboration strategy is used, whereby IP is shared with a broad research community. There might also be a hybrid strategy, wherein the IP framework applied is negotiated on a case-by-case basis. In any case, much ambiguity remains about the type of IP strategy most suitable, calling for transparency and explicitness in IP policies (34) (Figure 1). Ideally, public and private partners complement each other in many ways. Both the public partner, such as NGOs or international governmental organizations (IGOs), and industry deploy IP, trade, and rules of competition. However, there is a difference in their market, mission, and strategy. The public partner serves as balancing force for the competitive advantages of industry (35).

## Pricing Models to Facilitate Access to Medicines

Increased access to essential medicines can be established by prolonging patent terms whereby the revenues are reinvested for drug donations (24). However, the donor model (e.g., the Mectizan Donation Program) has also encountered criticism. Long-term drug donations are unsustainable due to a lack of infrastructure for technical, economic, and political support (35).

Especially the production of low-cost medicines and distribution thereof is challenging. Specific financing mechanisms stimulate price reductions of essential medicines leading to rapid uptake. The Global Fund’s Affordable Medicines Facility for Malaria, for example, is set up to provide significant subsidies to the private sector, as a large portion of people access medicines primarily through private markets (9). Furthermore, generic competitors and trade regulations are key to driving prices down, requiring involvement of multi-sectoral global governance agencies, such as the WTO.

Differential or tiered pricing means selling essential medicines in LMICs at prices below those in industrialized countries (36). In order to avoid parallel trade (export) of low-priced drugs to high-income countries, contracts including confidential rebates are used. This concept gained attention in the profit as well as non-profit sector since setting a product price to consumers’ willingness or ability to pay seems to be a profit maximizing strategy. At the same time, tiered pricing can increase consumers’ welfare by creating access to medicines. Yet tiered pricing does not guarantee a price that is equitable or affordable (37). In addition, generic prices are generally lower than tiered prices. Furthermore, tiered prices will unlikely be reduced in case of absence of competition, on the contrary, tiered prices may increase when competition does arise.

Off-patent competition results in prices below those in a tiered pricing setting. But tiered pricing may lead to anticompetitive effects when a very low tiered price discourages market entry by potential competitors. It is in situations of low demand and production capacity that tiered pricing by a single producer in developing countries may result in the widest access. Some difficulties remain with tiered pricing: what is the “lowest price possible,” what is a “fair price,” and how to negotiate if own production capacity is not favorable. In this sense, market segmentation needs to be considered both across and even within national markets (38).

However, income-related price discrimination and competition alone may not lead to affordable prices in low-income countries (39). Because of skewed income distributions in LMICs, drug prices with respect to mean per capita income are the highest in the poorest countries. Generic prices are below originator prices but because of lack of regulatory requirements for generic quality in LMIC, the latter is heterogeneous and uncertain to consumers. The optimal pricing strategy of a manufacturer is based on the type of product and on consumer’ perceptions and willingness to pay for that product with a particular quality. Procurement procedures rely on minimum quality standards. They also introduce originator and generic prices compared with the counterpart retail pharmacy prices and may reduce uncertainty related to quality, hence focusing competition on price (39).

## Conclusion

Several mechanisms, based on partnerships, IP, and pricing, are used to promote healthcare delivery and reduction of global health disparities. The mechanisms applied involve mostly existing drugs and devices that have lost their economic value in developed countries. Besides investigating how access mechanisms currently used could be enhanced, there is obviously a need to increase research for NTDs. Supported by strong leadership and actions at national or regional level, the United Nations (UN) commitments on the “25 by 25 goal,” i.e., a 25% reduction in premature NCDs mortality by 2025, can be achieved (40). Organizations such as WHO, WIPO, and WTO have a leading role in designing policies applicable to the various stakeholders. A critical mass of strong leaders in science, policy making, financing, and education are pivotal in building an innovation continuum.

## Author Contributions

HS and IH contributed to the design of the manuscript, performed the research, and wrote the manuscript.

## Conflict of Interest Statement

The authors declare that the research was conducted in the absence of any commercial or financial relationships that could be construed as a potential conflict of interest.

## Abbreviations

APOC, African Programme for Onchocerciasis Control; BMGF, Bill & Melinda Gates Foundation; CD, communicable diseases; DNDi, drugs for neglected diseases initiative; EC, European Commission; EFPIA, European Federation of Pharmaceutical Industries and Associations; ELF, European Lead Factory; GAVI, global alliance for vaccines and immunizations; GCP, good clinical practice; GSK, GlaxoSmithKline; IMI, innovative medicines initiative; IP, intellectual property; IGO, international governmental organizations; IPRs, IP rights; IPTK bank, intellectual property, technology and know-how bank; LMIC, low- and middle-income countries; MA, market authorization; mHealth, mobile health application; MMV, medicines for malaria venture; MPP, medicines patent pool; NCD, non-communicable diseases; NGO, non-governmental organizations; NTD, neglected tropical diseases; OWH, Institute for One World Health; PDP, product development partnership; PPP, public–private partnership; R&D, research and development; TPP, Trans-Pacific Partnership; TRIPS, agreement on trade-related aspects of intellectual property rights; TTIP, Transatlantic Trade and Investment Partnership; UN, United Nations; WHO, World Health Organization; WIPO, World Intellectual Property Organization; WTO, World Trade Organization.
